# Mapping methylation quantitative trait loci in cardiac tissues nominates risk loci and biological pathways in congenital heart disease

**DOI:** 10.1186/s12863-021-00975-2

**Published:** 2021-06-10

**Authors:** Ming Li, Chen Lyu, Manyan Huang, Catherine Do, Benjamin Tycko, Philip J. Lupo, Stewart L. MacLeod, Christopher E. Randolph, Nianjun Liu, John S. Witte, Charlotte A. Hobbs

**Affiliations:** 1 Department of Epidemiology and Biostatistics, School of Public Health, Indiana University Bloomington, 1025 E. Seventh Street, Bloomington, 47405 IN USA; 2 Hackensack-Meridian Health Center for Discovery and Innovation, Nutley, NJ 07110 USA; 3 Baylor College of Medicine, Houston, TX 77030 USA; 4 Arkansas Children’s Research Institute, Little Rock, AR 72202 USA; 5 University of California at San Francisco, San Francisco, CA 94158 USA; 6 Rady Children’s Institute for Genomic Medicine, San Diego, CA 92123 USA

**Keywords:** DNA methylation, Quantitative trait loci, Cardiac tissue, Bayesian co-localization, Mendelian randomization

## Abstract

**Background:**

Most congenital heart defects (CHDs) result from complex interactions among genetic susceptibilities, epigenetic modifications, and maternal environmental exposures. Characterizing the complex relationship between genetic, epigenetic, and transcriptomic variation will enhance our understanding of pathogenesis in this important type of congenital disorder. We investigated *cis*-acting effects of genetic single nucleotide polymorphisms (SNPs) on local DNA methylation patterns within 83 cardiac tissue samples and prioritized their contributions to CHD risk by leveraging results of CHD genome-wide association studies (GWAS) and their effects on cardiac gene expression.

**Results:**

We identified 13,901 potential methylation quantitative trait loci (mQTLs) with a false discovery threshold of 5%. Further co-localization analyses and Mendelian randomization indicated that genetic variants near the *HLA-DRB6* gene on chromosome 6 may contribute to CHD risk by regulating the methylation status of nearby CpG sites. Additional SNPs in genomic regions on chromosome 10 (*TNKS2-AS1* gene) and chromosome 14 (*LINC01629* gene) may simultaneously influence epigenetic and transcriptomic variations within cardiac tissues.

**Conclusions:**

Our results support the hypothesis that genetic variants may influence the risk of CHDs through regulating the changes of DNA methylation and gene expression. Our results can serve as an important source of information that can be integrated with other genetic studies of heart diseases, especially CHDs.

**Supplementary Information:**

The online version contains supplementary material available at 10.1186/s12863-021-00975-2.

## Background

Epigenetic modifications, such as DNA methylation, arise in response to internal and external stimuli and lead to metastable alterations of gene expression during cell development and proliferation, facilitating the adaptation of an individual cell to its environment [1]. DNA methylation patterns are known to vary substantially across individuals and tissue types, and can be associated with complex diseases and human traits, such as body mass index [2], cancer [3], diabetes [4] and birth defects [5]. However, the underlying mechanisms have not been comprehensively explored and are not fully understood. DNA methylation is known to influence various transcriptional processes, such as activation, repression, alternative splicing and genomic imprinting [6–8].

Importantly, DNA methylation has also been found to be genetically regulated [9]. A number of studies have identified genetic loci harboring sequence variants (mostly single nucleotide polymorphisms, SNPs) associated with quantitative changes in cytosine methylation levels in nearby CpG dinucleotides, referred to as methylation quantitative trait loci (mQTLs or meQTLs; here we utilize the first abbreviation) [10–12]. These mQTLs are primarily *cis*-acting and often co-localize with gene expression quantitative trait loci (eQTLs). These findings have provided a strong basis to hypothesize that causal genetic variants for complex diseases may function through regulating the methylation or expression level of genes within specific tissues. By this reasoning, mapping of mQTLs in disease-relevant human tissues, followed by overlapping such maps with genome wide association study (GWAS) data, can point to functional regulatory SNPs (rSNPs) that can affect disease risk [13, 14].

Congenital heart defects (CHDs) arise early in embryogenesis, during which epigenetic mechanisms are crucial in shaping a multitude of cell types and organs. Disruption of such control mechanisms may lead to a wide variety of diseases with behavioral, endocrine, or neurologic manifestations and disorders of tissue growth, including structural birth defects [15]. Several human developmental disorders, such as Prader-Willi, Angelman, Silver-Russell and Beckwith-Wiedemann syndromes, are known to be caused by epigenetic alterations, including loss or gain of imprinting (i.e. epimutations), uniparental disomy, or mutation/deletion of epigenetically regulated genes [16]. For CHDs, we and others have used maternal long interspersed nucleotide elements (LINE)-1 DNA methylation as a surrogate marker of global methylation, finding that maternal LINE-1 DNA hypo-methylation was associated with an increased risk of CHDs (OR = 1.91; 95% CI: 1.03, 3.58) [17, 18]. However, few studies have explored the functional effects of genetic variants on methylation patterns in human cardiac tissues.

Here we postulate that characterizing the complex relationship between genetic, epigenetic, and transcriptomic variation will provide insights into mechanisms of CHD. To pursue this hypothesis, we jointly analyzed genomic and epigenomic data to identify mQTLs within human fetal and adult cardiac tissue samples. We then refined these mQTL findings by leveraging results from our on-going GWASs of congenital heart defects, and subsequently prioritized genomic regions by co-localization analysis with GWAS findings and publicly available eQTL data.

## Results

### Identification of mQTLs

We conducted association tests for a total number of 30,774,423 SNP-CpG pairs that were within 75 KB distance. Benjamin-Hochberg false discovery rate was applied to adjust for multiple comparisons. The volcano plot and distribution of model goodness-of-fit (R^2^) are provided in Supplementary Fig.S1. After applying three pre-defined criteria of false discovery rate (< 0.05), regression coefficients (> 0.1 or < − 0.1) and goodness-of-fit (> 0.5), a total of 24,188 SNP-CpG pairs were identified, involving 1676 CpG sites and 13,901 SNPs as potential mQTLs. The results are available in the Supplementary Table S1. In all tables and figures, the genomic positions of SNPs and CpG sites were based on assembly GRCh37/hg19.

To provide additional insights into our mQTL findings, we first evaluated these potential mQTLs for their effects in the CHD genome-wise association studies (GWAS). Both of our GWAS phases had a case-parental trio design, including 440 and 225 trios, respectively. Each GWAS subject was genotyped by Illumina® Infinium HumanOmni5Exome BeadChip. Among the 13,901 SNPs identified as potential mQTLs, a total of 11,116 were tested in both phases of GWAS. We further found that 27 SNPs achieved nominal significance level in both GWAS phases.

**Fig. 1:**
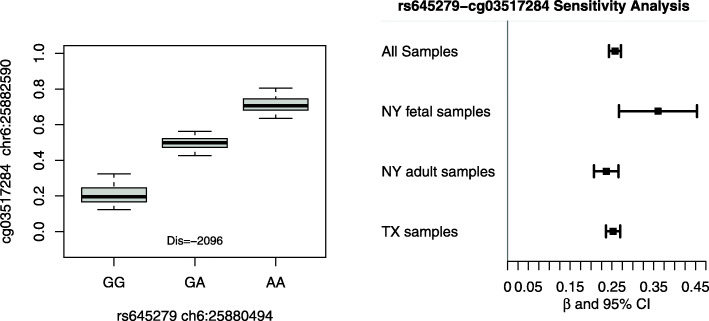
The genotypes of SNP rs645279 may influence the methylation of a CpG site cg03517284 that is located ~ 2000 base pairs away. Left: Distribution of methylation by SNP genotype. Right: sensitivity analysis of the association within all samples, NY fetal samples, NY adult samples and TX samples

The genotypes of these 27 SNPs appear to influence the methylation level of 11 CpG sites. We further limited the findings to regions that include at least 2 CpG sites within 2 KB or at least 2 SNPs within 75 KB, resulting in 25 SNPs and 9 CpG sites. The results are summarized in Table 1. A total of 21 SNPs were located on chromosome 6 forming 3 LD blocks, chr6: BP 25,874,823 – 25,888,643, BP 26,582,546 – 26,662,929 and BP 32,583,653 – 32,590,501. The remaining 4 SNPs were located on chromosome 7 (2 SNPs) and 8 (2 SNPs). We further examined how each individual SNP influenced the methylation level of its nearby CpG site. Figure 1A gives an example of an SNP rs645279 on chromosome 6 potentially regulating the methylation of a nearby CpG site cg03517284. To address the potential residual confounding effect from age and other unknown factors, we further conducted sensitivity analysis for the identified SNP-CpG pairs through stratified analyses within NY fetal samples, NY adult samples and TX samples. Figure 1B shows that the association between SNP rs645279 and CpG site cg03517284 was robust across three subpopulations. We hypothesize that the genotype of the mQTL SNPs may potentially influence the risk of CHD through regulating the methylation level of CpG sites. The methylation pattern by SNP genotype for other SNP-CpG pairs are provided in Supplementary Fig.S2. Among the 33 SNP-CpG pairs identified in Table 1, a total of 31 showed consistent direction of effect across 3 sub-populations. The results for all 33 SNP-CpG pairs are provide in Supplementary Fig.S3.

**Table 1:** SNPs identified as mQTLs that also achieved nominal significance level in both CHD GWASs

SNP ID	CHR	POSITION ^a^	CpG	POSITION	p.mQTL.BH	p.GWAS1	p.GWAS2
rs1165201	6	25,874,823	cg07061783	25,882,402	2.62e-16		
			cg03264133	25,882,463	1.57e-22	0.0364	0.0472
			cg03517284	25,882,590	5.42e-23		
rs645279	6	25,880,494	cg07061783	25,882,402	3.30e-22		
			cg03264133	25,882,463	2.62e-37	0.0167	0.0245
			cg03517284	25,882,590	9.79e-41		
rs112505305	6	25,888,643	cg07061783	25,882,402	1.92e-20		
			cg03264133	25,882,463	2.56e-30	0.0216	0.0133
			cg03517284	25,882,590	6.04e-30		
kgp3256684	6	26,584,526	cg06728252	26,598,149	1.70e-08	0.0075	0.0109
kgp10820427	6	26,590,801	cg06728252	26,598,149	8.16e-10	0.0017	0.0045
kgp12032951	6	26,597,893	cg06728252	26,598,149	8.16e-10	0.0024	0.0044
rs62394558	6	26,604,650	cg06728252	26,598,149	1.36e-06	0.0259	0.0024
kgp7659217	6	26,608,261	cg06728252	26,598,149	8.16e-10	0.0020	0.0045
kgp6693296	6	26,622,734	cg06728252	26,598,149	1.68e-08	0.0016	0.0054
rs2451731	6	26,624,822	cg06728252	26,598,149	1.68e-08	0.0017	0.0055
rs6552718	6	26,628,005	cg06728252	26,598,149	1.36e-06	0.0413	0.0053
rs1021372	6	26,632,444	cg06728252	26,598,149	1.68e-08	0.0026	0.0042
rs1021373	6	26,632,457	cg06728252	26,598,149	1.68e-08	0.0022	0.0054
rs2451744	6	26,633,463	cg06728252	26,598,149	1.61e-08	0.0032	0.0255
rs2494701	6	26,634,432	cg06728252	26,598,149	1.68e-08	0.0018	0.0063
kgp8313695	6	26,639,613	cg06728252	26,598,149	1.68e-08	0.0013	0.0041
kgp3537733	6	26,641,627	cg06728252	26,598,149	1.68e-08	0.0020	0.0055
rs116073375	6	26,650,826	cg06728252	26,598,149	1.68e-08	0.0020	0.0055
kgp4197236	6	26,662,920	cg06728252	26,598,149	1.68e-08	0.0023	0.0106
kgp4589793	6	32,583,653	cg19575208	32,551,888	9.87e-07	0.0192	0.0457
			cg24242384	32,551,954	3.35e-05		
rs9271573	6	32,590,501	cg08845336	32,551,891	1.24e-06	0.0038	0.0262
			cg24242384	32,551,954	9.14e-06		
rs10953985	7	123,488,985	cg09630417	123,459,295	3.29e-06	0.0359	0.0244
rs10276917	7	123,498,400	cg09630417	123,459,295	3.29e-06	0.0359	0.0255
rs6996562	8	33,392,023	cg20849935	33,432,330	0.001062	0.0495	0.0416
rs9297205	8	33,407,400	cg20849935	33,432,330	2.03e-06	0.0207	0.0417

^**a**^ Genomic position based on assembly GRCh37/hg19 for all tables and figures

### Bayesian co-localization analysis

**Fig. 2:**
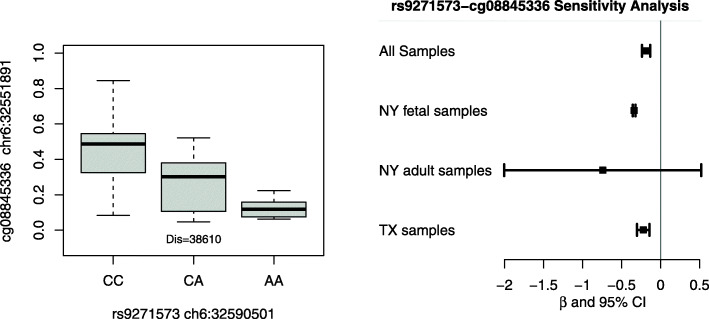
The genetic-epigenetic association between SNP rs9271573 and cg08845336. The SNP lies within a HLA region that colocalized with a gene expression QTL, and achieves nominal statistical significance in both phases of CHD GWAS

To leverage results from other data sources, we further conducted co-localization analysis between mQTL results and CHD GWAS results and eQTL results from GTEx database. Our goal was to prioritize regions with high probability (i.e. PP4 > 0.95) supporting H4: there exists a single causal variant common to both traits. The results are summarized in Table 2, and the distributions of all posterior probabilities for H0 – H4 are illustrated in Supplementary Fig.S4. In particular, one region on chromosome 6 (BP 32,583,653 – 32,590,501) in Table 1 was very close to the gene unit (*HLA-DRB6*; BP: 32,520,489 – 32,527,779) and was identified by colocalization analysis between mQTL and GWAS phase 1 results, indicating shared causal genetic variants within the region that regulate both DNA methylation and CHD risk. SNP rs9271573 has a minor allele frequency of 42.7% in our study, which is in line with the reported allele frequencies from dbSNP (41.3–47%). The genetic-epigenetic association between rs9271573-cg08845336 and its sensitivity analysis is illustrated in Fig. 2. In addition, six gene units were identified to have shared causal variants for both methylation level and gene expression levels in two types of cardiac tissues. For example, chromosome 10 (*TNKS2-AS1*; BP: 93,542,595 – 93,558,048) may harbor both a mQTL and an eQTL within four types of cardiac tissues, “Artery Aorta”, “Artery Tibial”, “Heart Artial Appendage” and “Heart Left Ventricle”. Chromosome 14 (*LINC01629*; BP: 77,425,980 – 77,432,145) may harbor both a mQTL and an eQTL within three types of cardiac tissues, “Artery Aorta”, “Artery Tibial” and “Heart Artial Appendage”. The other four regions, including chromosome 5 (*TARS*; BP: 33,440,801 – 33,468,196), chromosome 10 (*BORCS7/AS3MT*; BP: 104,613,966 – 104,661,655), chromosome 19 (*HKR1*; BP: 37,803,738 – 37,855,358) and chromosome 19 (*ZNF320*; BP: 53,362,743 – 53,400,947), were identified to colocalize with an eQTL within two types of cardiac tissues.

**Table 2:** Co-localization analysis with two CHD GWASs and eQTL findings

Chro	Regions	Gene	Source for Co-localization	PP0	PP1	PP2	PP3	PP4
6	32,520,489–32,527,779	*HLA-DRB6*	GWAS – Phase 1	1.12e-10	0.028	1.95e-09	0	0.972
10	93,542,595–93,558,048	*TNKS2-AS1*	eQTL – Artery Aorta	1.11e-02	2.11e-02	5.07e-04	0	0.967
			eQTL – Artery Tibial	2.12e-03	4.04e-03	5.21e-04		0.993
			eQTL – Heart Atrial Appendage	1.34e-04	2.56e-04	5.24e-04		0.999
			eQTL – Heart Left Ventricle	8.05e-04	1.53e-03	5.23e-04		0.997
14	77,425,980–77,432,145	*LINC01629*	eQTL – Artery Aorta	3.70e-03	2.95e-02	1.97e-04	6.04e-04	0.966
			eQTL – Artery Tibial	1.38e-07	1.09e-06	1.27e-04	0	0.999
			eQTL – Heart Artial Appendage	3.11e-06	2.45e-05	1.27e-04	0	0.999
5	33,440,801–33,468,196	*TARS*	eQTL – Artery Aorta	2.72E-07	1.37e-02	1.95e-08	0	0.986
			eQTL – Artery Tibial	2.41E-08	1.22e-03	1.97e-08	0	0.999
19	37,803,738–37,855,358	*ZNF875*	eQTL – Heart Artial Appendage	1.93e-05	5.59e-07	3.34e-02	0	0.967
			eQTL – Heart Left Ventricle	2.42e-05	7.01e-07	3.34e-02	0	0;967
19	53,362,743–53,400,947	*ZNF320*	eQTL – Artery Aorta	1.03e-12	2.85e-03	3.50e-14	0	0.972
			eQTL – Artery Tibial	6.95e-13	1.93e-03	3.53e-14	0	0.981
10	104,613,966–104,661,655	*BORCS7 / ASMT*	eQTL – Artery Aorta	1.10e-02	6.92e-04	1.55e-02	5.11e-06	0.973
			eQTL – Artery Tibial	7.95e-05	5.00e-06	1.57e-02	0	0.984

PP0 – PP4: Bayesian posterior probability for hypotheses H0 to H4, respectively

H0: there exist no causal variants for either trait;

H1: there exists a causal variant for trait 1;

H2: there exists a causal variant for trait 2;

H3: there exist two distinct causal variants, one for each trait; or

H4: there exists a single causal variant common to both traits

*HLA-DRB6*: major histocompatibility complex, class II, DR beta 6

*TNKS2-AS1*: TNKS2 antisense RNA 1

*LINC01629*: long intergenic non-protein coding RNA 1629

*TARS*: threonyl-tRNA synthetase

*ZNF875*: *Homo sapiens* zinc finger protein 875

*ZNF320:* zinc finger *protein 320*

*BORCS7 / ASMT*: BLOC-1 related complex subunit 7 / acetylserotonin O- methyltransferase

### Mendelian randomization

**Fig. 3:**
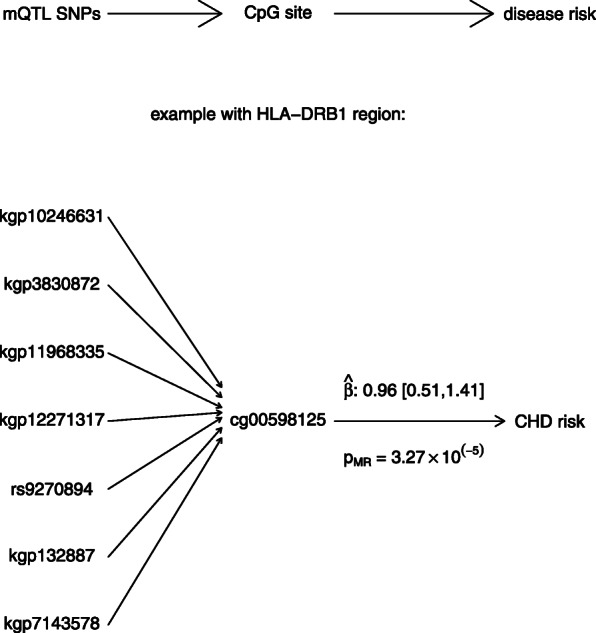
Two-sample Mendelian randomization based on mQTL results and CHD GWAS2 identified a CpG site (cg00598125) close to HLA genes for influencing CHD risk

As described above, a total of 1676 CpG sites were associated with one or more mQTL SNPs. For each CpG site, we used its associated mQTLs as instrumental SNPs and conducted two-sample Mendelian randomization with each of the CHD GWASs. We were able to conduct analysis for 1316 and 1275 CpG sites that had at least one instrumental SNP available in GWAS phase 1 and phase 2, respectively. After Bonferroni correction for 1316 tests (i.e. threshold of 3.80e-05), a total of 12 CpG sites were identified with potential causal effect on CHD risk. The results are summarized in Supplementary Table S2. In particular, one CpG site, cg00598125, was located at chr6: BP 32,555,411 (Table 3). This CpG site was very close to a genomic region identified by co-localization analysis in Table 2 (chr6: BP 32,583,653 – 32,590,501), which overlapped with the region of its 7 instrumental SNPs (chr6: BP 32,504,218 – 32,589,959). The hypothesized causal pathway and estimated causal effect of cg00598125 on CHD risk is presented in Fig. 3. More detailed results for other CpG sites are provided in Supplementary Fig.S5. As discussed in method section, this MR analysis is exploratory with required assumptions. However, these results support our hypothesis that mQTL SNPs may influence the risk of CHD through regulating the methylation of CpG sites.

**Table 3:** Mendelian Randomization for the causal effect of CpG sites on CHD risk

CpG	CHR	POSITION	Nearby Gene	mQTL SNP^a^	POSITION	p.MR1^b^	p.MR2^c^
cg00598125	6	32,555,411	*HLA-DRB1*	kgp10246631	32,504,218	0.53	3.27e-05
				kgp3830872	32,519,391		
				kgp11968335	32,554,197		
				kgp12271317	32,561,327		
				rs9270894	32,571,872		
				kgp132887	32,578,970		
				kgp7143578	32,589,959		

^**a**^ 7 mQTL SNPs of cg00598125 were used as instrumental SNPs in Mendelian randomization

^b^ MR analysis in CHD GWAS Phase 1

^c^ MR analysis in CHD GWAS Phase 2

To compare with eQTL findings, we also conducted two-sample MR using GTEx and CHD GWASs to evaluate the potential causal effect of gene expression on CHD risk. While no genes were statistically significant after Bonferroni adjustment, one gene (*LMO7*) on chromosome 13 achieved the nominal significance level via MR-eQTL analysis using an eQTL instrumental SNP (i.e. rs9318373). A CpG site within gene *LMO7* (i.e. cg02349334) was also identified via MR-mQTL analysis using 5 mQTL instrumental SNPs (Table 4).

**Table 4:** One gene identified by MR-mQTL analysis and achieved the nominal significance level in MR-eQTL analysis

	Exposure ^a^	CHR	POSITION	Nearby Gene	Instrumental SNP ^b^	POSITION	p.MR1^c^
MR-mQTL	cg02349334	13	76,363,851	*LMO7*	rs530855	76,337,780	3.92e-07
					rs9318373	76,363,721	
					rs660942	76,373,924	
					kgp9606293	76,391,057	
					rs9600564	76,435,635	
MR-eQTL	*LMO7*	13	76,194,570–76,434,006	*LMO7*	rs9318373	76,363,721	8.27e-03

^**a**^ MR-mQTL evaluated the causal effect of CpG site on CHD risk, while MR-eQTL evaluated the causal effect of gene expression on CHD risk

^b^ 5 mQTL SNPs and 1 eQTL SNP was avaiable for MR-mQTL and MR-eQTL, respectively

^c^ MR analysis in CHD GWAS Phase 1

### Comparison with findings in the literature

**Fig. 4:**
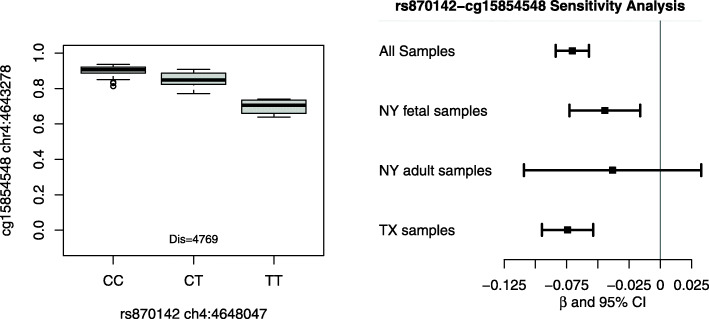
DNA methylation of CpG site cg15854548 associated with SNP rs870142. SNP rs870142 was identified by GWAS for association with atrial septal defects (ASDs). It was located at chromosome 4p16, and replicated in two independent studies for association with ASDs

Several GWASs have been conducted for CHDs in the literature [19–27]. However, their top findings have not been consistent across studies [28]. We further looked into those GWAS identified SNPs for association with the methylation levels at nearby CpG sites in our samples. A total of 8 SNPs were available in our data with at least 3 samples in each genotype group and at least 1 CpG site within 75 KB distance. These 8 SNPs led to a total of 138 SNP-CpG pairs, and the association results are available in Supplementary Table S3. One SNP-CpG pair (rs870142 - cg15854548) achieved statistical significance at false discovery rate of 5% (*p*-value = 8.96e-16). The methylation distributions of cg15854548 by the genotype of rs870142 is illustrated in Fig. 4. In particular, SNP rs870142 was found to be associated with atrial septal defects (ASDs) in an European population [19]. It was located at chromosome 4p16, and was independently replicated in two studies of ASDs in Han Chinese populations [29, 30]. In our study, SNP rs870142 was not identified as mQTL because the regression coefficients was less than the pre-defined threshold of 0.1 (i.e. *β* = 0.07). However, it would be interesting for additional studies to evaluate its involvement to regulate DNA methylation.

## Discussion

We presented a study of the genetic effects on DNA methylation within cardiac tissues with the goal to enhance our understanding of the complex mechanism underlying the development of CHDs. We thus prioritized the genomic regions by leveraging findings from GWAS and tissue-specific eQTLs. We showed that a few genomic regions may potentially harbor genetic variants that simultaneously influence DNA methylation, gene expression, or CHD risk. Recent studies suggested that genetic contribution to CHD may be mediated through transcriptional and post-transcriptional regulatory effects during cardiac development [31]. Our results add to an increasing body of evidence showing that the genetic influences on DNA methylation are widespread across the genome [32], and suggest that the risk of CHD may be genetically mediated through the changes of DNA methylation and gene expression. To our knowledge, our study is among the first to investigate the genetic architecture of DNA methylation within cardiac tissue samples on a genome-wide and epigenome-wide scale and its contribution to CHD risk. Our results can serve as an important source of information that can be integrated with other genetic studies of heart diseases, especially CHDs.

Our findings provide novel insight into our understanding of the etiology of CHDs, especially the identified genomic regions and gene units with multiple sources of evidence supporting their biological plausibility. In particular, gene *HLA-DRB6* (major histocompatibility complex, class II, DR beta 6) is one of the human major histocompatibility complex (MHC) genes. Our study found that this gene may be implicated in both genetic-epigenetic association and CHD GWAS. A previous study found that this gene was significantly associated with gestational diabetes mellitus among pregnant women [33]. Maternal diabetes may increase the risk of various congenital anomalies, including CHDs [34, 35]. Previous studies using NBDPS samples found that gestational diabetes was associated with three cardiac malformations, including tetralogy of Fallot, pulmonary valve stenosis, and atrial septal defect [35]. It was estimated that CHDs occur in 5% of infants of diabetic mothers, and most frequently if the mother has gestational diabetes and develops insulin resistance in the 3rd trimester [36].

A number of gene units were identified to harbor genetic variants that may regulate both DNA methylation and gene expression. Gene *TNKS2-AS1*, or Tankyrases 2 antisense RNA 1, is located on chromosome 10, and approximately 100 bps upstream of gene *TNKS2* (BP: 93,558,151 – 93,625,232). A previous study suggested that structure changes within *TNKS2-AS1* was linked with dysregulation of gene expression in dilated cardiomyopathy [37]. Studies have also found that tankyrases were involved in various cellular functions, such as metabolic homeostasis, telomere length maintenance, cell cycle progression and heritable disease cherubism [38–41]. Animal studies have found that *TNKS2* was essential for embryonic development and normal growth [42, 43]. Another gene unit, *LINC01629*, or long intergenic non-protein coding RNA 1629, is located on chromosome 14. The GTEx project identified the region as a potential eQTL in “Artery Tibial” and “Heart Artial Appendage” [44]. Another study with RNAseq data analysis further found that the expression level was biased in heart and placenta [45], indicating its functional implication in both heart and during pregnancy. The results of our study are consistent with existing findings, and further suggests that the methylation changes may also be involved. It is also biologically plausible that DNA methylation plays an important role in regulating its gene expression in heart tissues contributing to the CHD development.

Our study must be considered in the light of certain limitations. First, the sample size of our study is relatively small, which is largely due to the difficulty in collecting cardiac tissue samples. As a result, our analysis was limited to common variants with at least three samples for each genotype group. Second, the tissue samples were collected at different locations and were not available to us at the same times, which increase the chances for confounding bias. In our analysis, we have tried to minimize the impact by normalizing raw data together and adjusting for the top principal components of both genomic and epigenomic profiles. Third, our analysis was based on the association between each single SNP and single CpG site. No possible gene-by-gene interactions or gene-by-environment interactions have been considered. Forth, while prioritizing our mQTL findings with existing knowledge, we used a commonly used package, *coloc*, for colocalization analysis. Additional methods have been recently proposed with improvements. For example, the enrichment estimated aided colocalization analysis (*enloc*) and *fastenloc* were able to integrate enrichment analysis with colocalization analysis [46, 47]. It also uses the deterministic approximation of posteriors (DAP) algorithm for Bayesian multi-SNP fine mapping and genomic annotation. Further analysis with additional strategies may yield additional findings [48]. Fifth, the genetic causes of CHDs are largely unclear. When prioritizing the findings for CHD risks, we are limited by the existing knowledge of the genetic etiology of CHDs. Very few GWASs have been conducted for CHDs, and the sample sizes are relatively small compared to studies of other complex human diseases. We have used a nominal threshold of 0.05 while leveraging our CHD GWAS results in order to provide plausible candidates to be evaluated by well-powered GWASs in the future.

## Conclusions

We have identified mQTLs within cardiac tissue samples, and prioritized our findings by leveraging results from other sources, including GWAS and eQTL database. Our results suggest that genetic variants near the *HLA-DRB6* gene on chromosome 6 may contribute to CHD risk by regulating the methylation status of nearby CpG sites. Additional SNPs in genomic regions on chromosome 10 (*TNKS2-AS1* gene) and chromosome 14 (*LINC01629* gene) may simultaneously influence epigenetic and transcriptomic variations within cardiac tissues. Our results support the hypothesis that genetic variants may influence the risk of CHDs through regulating the changes of DNA methylation and gene expression.

## Methods

### Study population

Our study includes cardiac tissue samples from 87 patients from three states, including New York (NY; *n* = 33; 15 fetal and 18 adult), Texas (TX; *n* = 50; ages < 19 years) and Arkansas (AR; *n* = 4; ages unknown). The NY samples were collected through the autopsy service at Columbia University, and were from fetal and adult cases without known heart diseases. The TX samples were collected at Texas Children’s Hospital/Baylor College of Medicine, and were from subjects who were diagnosed with CHDs and underwent surgical intervention. Specifically, these tissues were obtained during surgical repair of the CHD and stored in the Research and Tissue Support Services (RTSS) core at Texas Children’s Hospital. The AR samples were collected by the Arkansas DNA Bank for Congenital Malformations funded by Arkansas Reproductive Health Monitoring System. Cardiac tissues were excised during surgical repair of structural heart defects, flash frozen at time of OR, retrieved by research nurse in Eppendorf tubes, transported in liquid nitrogen portable container, and then stored in liquid nitrogen at Arkansas Children’s Research Institute. After the quality control process described below, three samples were removed because of low genotype call rate, and one sample was removed because of abnormal distribution of epigenomic profile (described below). A total number of 83 samples remained for analysis.

### Genomic and Epigenomic profiling

Tissue samples from TX and AR were processed at the Center for Translational Pediatric Research Genomics Core Lab at the Arkansas Children’s Research Institute. Samples from New York were received as purified DNA. Human heart tissue was stored in liquid nitrogen (vapor phase) until it was processed. The MP FastPrep-24 5G instrument (MP Biomedicals) and MP Fast DNA Spin kit for Plant and Animal Tissue (MP Biomedicals) were used to homogenize and lyse sample tissue (approximately 30 mg) and isolate and purify DNA following the manufacturers instrument and kit protocol. Genomic DNA was quantified by use of a Qubit fluorometer and Qubit dsDNA HS assay kit (Invitrogen). All genetic and epigenetic profilings were conducted at Arkansas Children’s Research Institute to minimize the technical variations.

For genetic data, all samples were genotyped for approximately 5 million SNPs using Illumina® Infinium HumanOmni5Exome BeadChip. Illumina’s detailed protocol was followed to process 200 ng DNA samples through Infinium processing, resulting in genotype-dependent fluorescent signals that were detected using Illumina software on an Illumina iScan platform. Data and images produced by the scanner were transferred in real time to the Images server at University of Arkansas for Medical Sciences. Illumina’s GenomeStudio was used for initial genotype calling and assay quality check.

For epigenomic data, the NY and AR samples were profiled using Illumina® Infinium HumanMethylation450 BeadChips, which interrogate > 450 K methylation sites, including regulatory elements such as promoter-associated CpG islands, non-island methylated sites including enhancer and insulator elements, and miRNA promoter regions. As the tissues from TX were subsequently obtained, these samples were profiled using Illumina® Infinium MethylationEPIC BeadChips, which interrogate approximately 850 K potentially methylated CpG sites. All samples were processed following the standard protocol provided by Illumina™ for DNA methylation analysis. Bisulfite modification of 500 ng of genomic DNA was accomplished by use of the EZ DNA Methylation-Direct Kit (*Zymo Research*, Orange, CA). The bisulfite converted DNA was resuspended in 12 μl TE buffer and stored at − 80 °C until the samples were ready for analysis. Further, 4 μl of bisulfite converted DNA was isothermally amplified at 37 °C overnight. The amplified DNA product was fragmented by an end point enzymatic process, then precipitated, resuspended, and applied to Illumina Infinium® BeadChip for overnight hybridization. During hybridization, the amplified and fragmented DNA samples annealed to specific oligomers which were covalently linked to different bead types. Each bead type corresponded to the nucleotide identity and thus reflected the methylation status at a bisulfite converted cytosine in a specific CpG site. The bead chips were then subjected to a single base extension reaction using the hybridized DNA as a template, incorporating fluorescently labeled nucleotides of two different colors, corresponding to the cytosine (methylated) or uracil (unmethylated) identity of the bisulfite converted nucleotide at a specific CpG site. The fluorescently stained chip was imaged on an Illumina iScan.

### Data processing and quality control

For *epigenomic* data, we used the Bioconductor package “*minfi*” in R to combine the raw intensity values from all samples at the same time [49–51]. Functional normalization was applied to raw intensities, which used internal control probes on each array to remove between-array technical variations. We only considered overlapping CpG sites between the HumanMethylation450 BeadChip and MethylationEPIC BeadChip. Beta values were produced to measure the methylation level of CpG sites, and intensities with detection *p*-values greater than 0.01 were set to missing. We further removed CpG sites with more than 5% missing values or with a SNP in the probe. After the data processing, a total of 435,525 CpG sites remained for further analysis.

For *genomic data*, we used PLINK 1.9 for data processing [52, 53]. We removed samples with call rates less than 95% (*n* = 3), and further removed SNPs if they 1) had call rates less than 95%; 2) were located more than 75 KB away from any CpG site; 3) had minor allele frequencies below 5%; 4) deviated from Hardy-Weinberg equilibrium among control samples (*p*-value < 0.0001). After the data processing, a total of 1,659,340 SNPs remained for further analysis with an average call rate of 99.8%.

We used several procedures to ensure our data quality among 84 samples. First, we examined the log median intensity values in both methylated and unmethylated channels, as well as the density plot of beta values (Supplementary Fig.S6 Panels A and B). Both figures suggest that the overall distributions were relatively consistent across samples after normalization. Only one sample showed major deviation from the group in Supplementary Fig.S2 (internal sample ID: NY07). Second, we conducted a sex check for both genomic and epigenomic data. Specifically, sex was inferred by both genomic data and epigenomic data separately, and was 100% consistent between the two platforms, resulting in 39 male samples and 45 female samples. Third, we conducted principal component (PC) analysis and evaluated the clustering of samples based on the top 4 PCs (Supplementary Fig.S6 Panel C). One sample largely deviated from the others (internal sample ID: NY07), and was the same sample identified by density plot of beta values described above. We therefore removed this sample from further analysis. Samples from three states showed differences especially with respect to the first PC. We did not have the age information of most of our samples. However, we were aware that NY samples included a mixture of fetal samples and adult samples, and TX samples were all from children under age of 19. The implied age of the samples showed differences especially with respect to the second PC. We did not observe any clustering pattens of samples for the additional PCs. Therefore, in the final analysis to detect mQTLs, we controlled for the top 5 PCs for both genomic and epigenomic data in order to adjust for the potential batch effect and other unknown confounding factors. For our top findings, we also conducted sensitivity analysis through stratified analyses within NY fetal samples, NY adult samples and TX samples.

### Identification of mQTLs

The final analytical dataset included cardiac tissues from 83 samples. Each sample had 1,659,340 SNPs and 435,525 CpG sites. We focused on the detection of *cis-* mQTLs, and conducted linear regression to evaluation the genetic-epigenetic association for all possible SNP and CpG pairs within 75 KB distance. We also adjusted for the case control status of CHD, sex, top 5 PCs of genomic data, and top 5 PCs of epigenetic data.

$$ \beta\ value\sim Genotype+ Disease+ Sex+{\sum}_{i=1}^5{PC}_i^{(g)}+{\sum}_{j=1}^5{PC}_i^{(e)}+\upvarepsilon; $$

where the SNP genotypes were coded as the minor allele counts. We further defined a SNP as a potential mQTL if all of the followings were met: 1) the genetic-epigenetic association was statistically significant at a false discovery rate of 0.05; 2) the regression coefficient for genotype effect on methylation level had an absolute value greater than 0.1; and 3) the regression model had a goodness-of-fit R-square great than 0.5. The rationale of choosing such criteria is detailed elsewhere [13].

### Bayesian co-localization

We further conducted co-localization analysis to leverage results from genome-wide association studies of CHDs and expression QTLs. Under co-localization analysis, each genomic locus was evaluated across two traits (i.e. methylation level and CHD status, or methylation level and gene expression level) by calculating the posterior probability for five hypotheses, with H4 as our main hypothesis of interests.

H0: there exist no causal variants for either trait;

H1: there exists a causal variant for trait 1;

H2: there exists a causal variant for trait 2;

H3: there exist two distinct causal variants, one for each trait; or.

H4: there exists a single causal variant common to both traits.

We and others have conducted two phases of GWAS for CHDs using samples from the National Birth Defects Prevention Studies (NBDPS). We thus considered results from three additional data sources: 1) CHD trait in GWAS Phase 1; 2) CHD trait in GWAS Phase 2; and 3) heart-tissue gene expression in Genotype-Tissue Expression (GTEx) database [44]. For eQTL results, five types of cardiac tissues were considered, including “Artery Aorta”, “Artery Coronary”, “Artery Tibial”, “Heart Atrial Appendage”, and “Heart Left Ventricle”. Each of the data sources was analyzed together with the mQTL results for co-localization. To identify biologically meaningful loci, we used UCSC Genome Browser (assembly GRCh37/hg19) to define gene units as candidate loci for co-localization analysis [54]. A candidate locus was defined as 7.5 KB upstream and downstream the corresponding gene region. Software *bedtools* were further used to extract the genomic regions based on the gene annotation [55]. After the gene extraction, a total number of 21,903 regions were considered as candidate loci. Bioconductor package “*Coloc*” was used for colocalization analysis [56–58].

### Mendelian randomization (MR)

Recently, MR has become a popular way to access causal effects using genetic variants as instrumental variables. To explore the underlying causal pathway between mQTL SNPs, CpG sites and CHD risk, we further performed two-sample MR by using the effect sizes of the identified mQTL SNPs on CpG sites and their corresponding effects in each of the CHD GWAS. The analysis was conducted by “*TwoSampleMR*” package in R [59]. The analysis had two main steps. First, we used software *haploview* [60] to select tag SNPs among mQTLs as “independent” instrumental variables for each CpG site involved. Second, a Wald ratio was calculated between the effect of an mQTL SNP on CHD risk and its effect on DNA methylation to evaluate the causal relationship between the CpG site and CHD risk. When multiple mQTLs were selected for one CpG site, inversely variance weighting was used to integrate the effects and heterogeneity test was conducted, given that the test of pleiotropy via Egger regression was not statistically significant [61].

It should also be noted that this MR analysis is exploratory, and relies on a few required assumptions. First, the selected mQTL SNPs are associated with the methylation level at the CpG site. Second, the selected mQTL SNPs are assumed to be independent of CHD risk given the CpG site and all other confounders. Third, the selected mQTL SNPs are assumed independent of other factors that may confound the relationship between the CpG site and CHD risk. Our data supports the first assumption by identifying mQTLs for CpG sites. When multiple mQTL SNPs were available, the test of pleiotropy via Egger regression showed no evidence of violating the second assumption. However, as a frequently noted limitation for MR, we have not been able to verify the last assumption.

## Supplementary Information

**Additional file 1: Figure S1 .** Distribution of modeling fitting statistics evaluating genetic-epigenetic association. Left: Volcano plot of coefficient estimates. Right: model goodness-of-fit R^2^.

**Additional file 2: Figure S2.** Distribution of methylation by the genotypes of mQTL SNPs in Table 1.

**Additional file 3: Figure S3.** Sensitivity analysis by subgroup analysis within NY fetal samples, NY adult samples and TX samples.

**Additional file 4: Figure S4.** Distribution of posterior probabilities (PP0 – PP4) from colocalization analysis with two GWAS phases and five eQTL heart tissues.

**Additional file 5: Figure S5.** Two-sample MR for causal effect from mQTL to CHD through CpG.

**Additional file 6: Figure S6.** Sample QC. **A.** Median intensities for methylated channels vs unmethylated channels. No bad quality sample was identified. **B.** Distribution of methylation beta values across samples. One sample (internal ID: NY07) showed abnormal distribution. **C.** Principal components of Epigenomic profiles. Red color: fetal heart samples; Green color: adult heart samples; Black color: ages unknown. One sample (NY07) was removed from the analysis.

**Additional file 7: Table S1.** List of mQTL identified. Available for download at https://github.com/liming81/Heart_mQTL.

**Additional file 8: Table S2.** CpG sites identified with potential causal effect on CHD risk by Two-sample Mendelian Randomization. Available for download at https://github.com/liming81/Heart_mQTL.

**Additional file 9: Table S3.** Association between GWAS identified SNPs (Lupo et al. 2019) and nearby CpG sites. Available for download at https://github.com/liming81/Heart_mQTL.

## Data Availability

The expression quantitative trait loci (eQTL) data used in this study is publicly available from the Genotype-Tissue Expression (GTEx) project at https://www.gtexportal.org/home/ (dbGaP Accession phs000424.v8.p2). The genetic and epigenetic datasets generated and analysed during the current study are not publicly available due to restrictions of protected health information but are available from the corresponding author subject to appropriate IRB approval. The R codes for the analyses were deposited at GitHub (https://github.com/liming81/Heart_mQTL).

## Abbreviations

CHDs: Congenital heart defects
SNP: Single nucleotide polymorphism
GWAS: Genome-wide association study
mQTL: Methylation quantitative trait locus
eQTL: Expression quantitative trait locus
rSNP: Regulatory SNP
LINE 1: Long interspersed nucleotide elements 1
NBDPS: National Birth Defects Prevention Studies
GTEx: Genotype-Tissue Expression
MR: Mendelian Randomization
ASD: Atrial septal defects
MHC: Major histocompatibility complex
NY: New York
TX: Texas
AR: Arkansas
RTSS: Research and Tissue Support Services
PC: Principal component
