# Supplementary figures and images for: Mapping methylation quantitative trait loci in cardiac tissues nominates risk loci and biological pathways in congenital heart disease
Source: BMC Genom Data. 2021 Jun 10;22:20. doi: 10.1186/s12863-021-00975-2 (PMC8194170; doi:10.1186/s12863-021-00975-2)

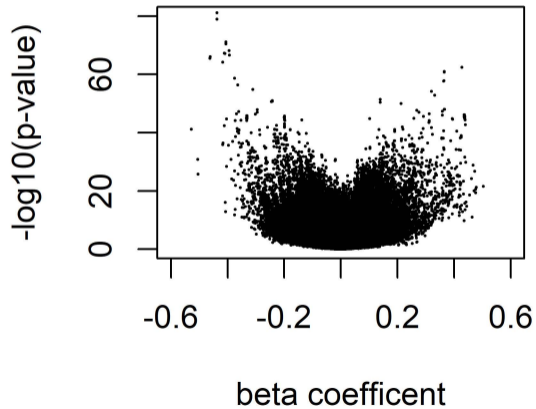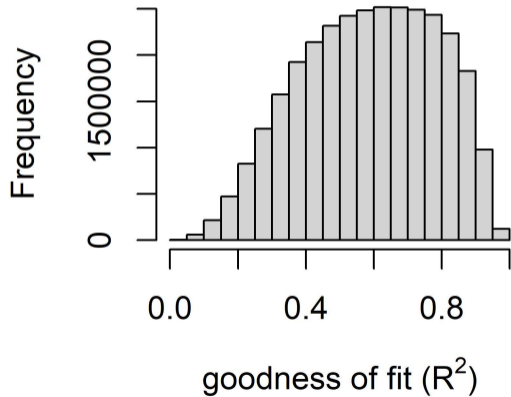

Supplement: Supplementary file 1 — Additional file 1: Figure S1 . Distribution of modeling fitting statistics evaluating genetic-epigenetic association. Left: Volcano plot of coefficient estimates. Right: model goodness-of-fit R2. [file 12863_2021_975_MOESM1_ESM.pdf]

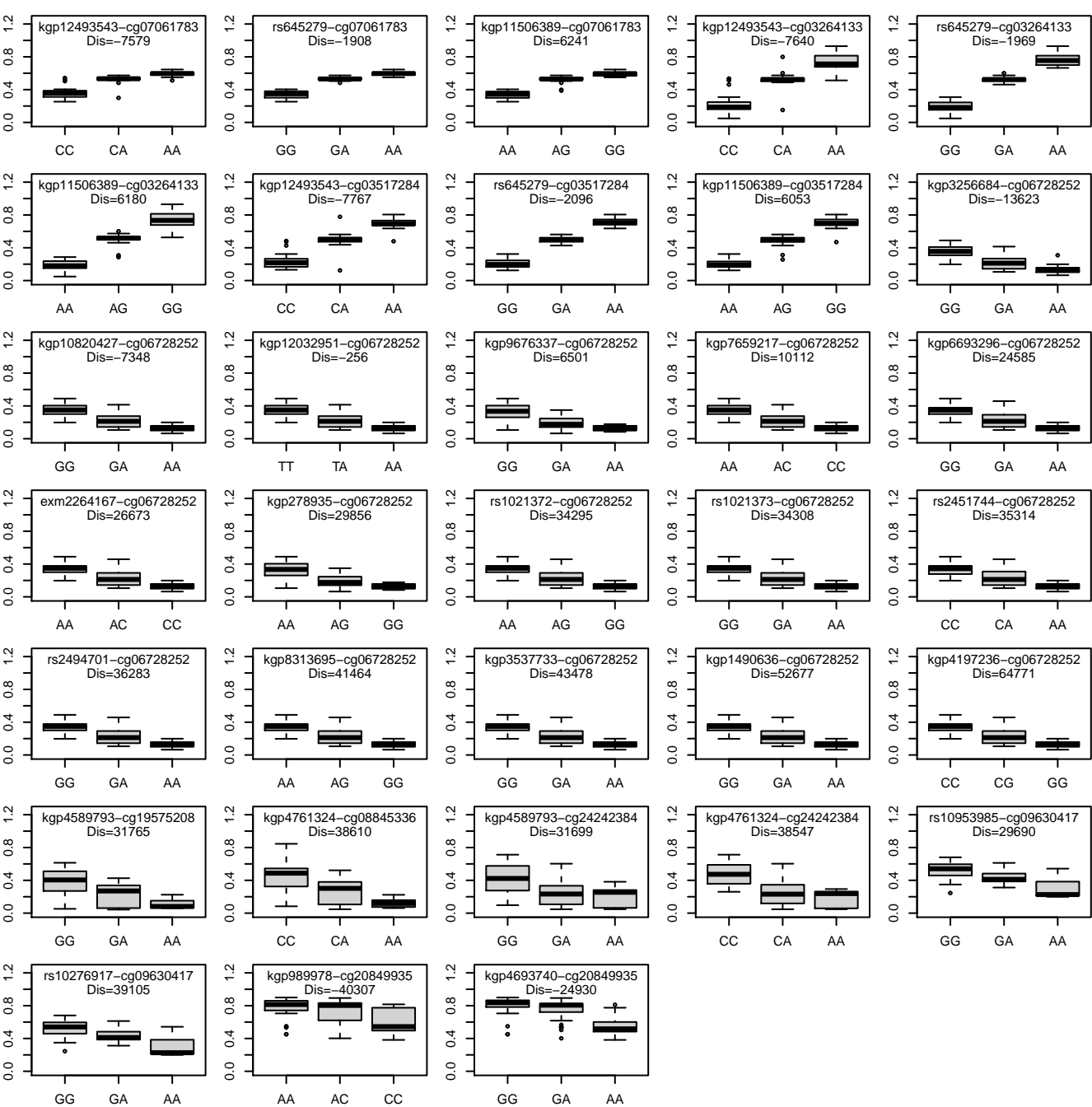

Supplement: Supplementary file 2 — Additional file 2: Figure S2. Distribution of methylation by the genotypes of mQTL SNPs in Table 1. [file 12863_2021_975_MOESM2_ESM.pdf]

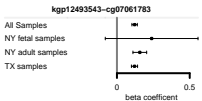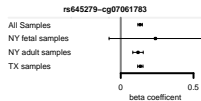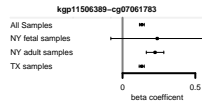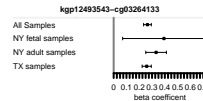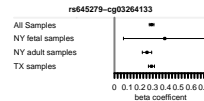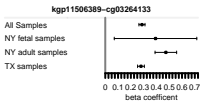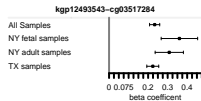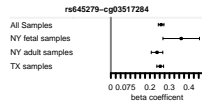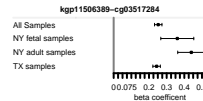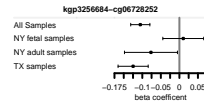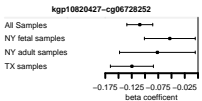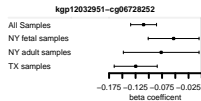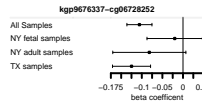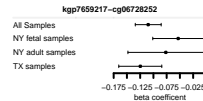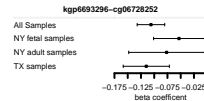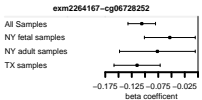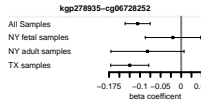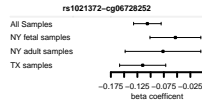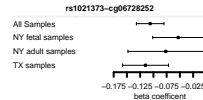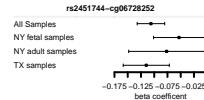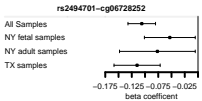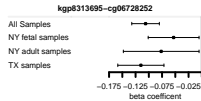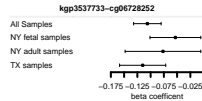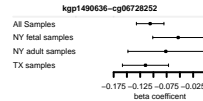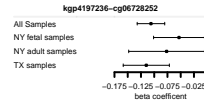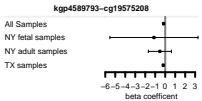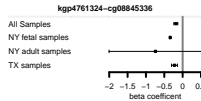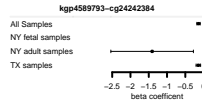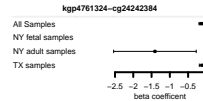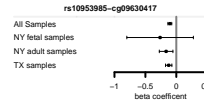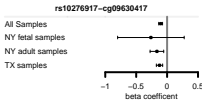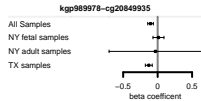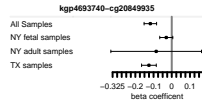

Supplement: Supplementary file 3 — Additional file 3: Figure S3. Sensitivity analysis by subgroup analysis within NY fetal samples, NY adult samples and TX samples. [file 12863_2021_975_MOESM3_ESM.pdf]

PP0

PP1

PP2

PP3

PP4

GWAS1

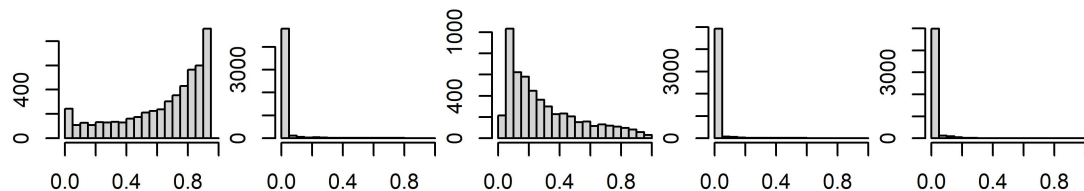

GWAS2

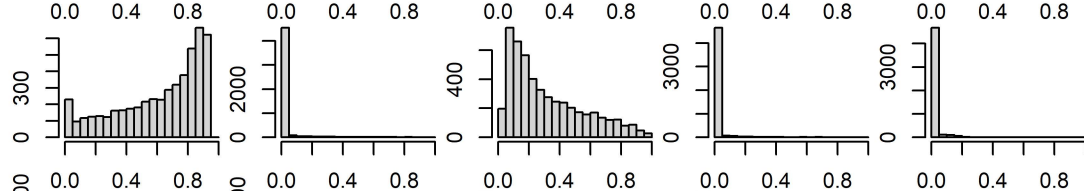

AA

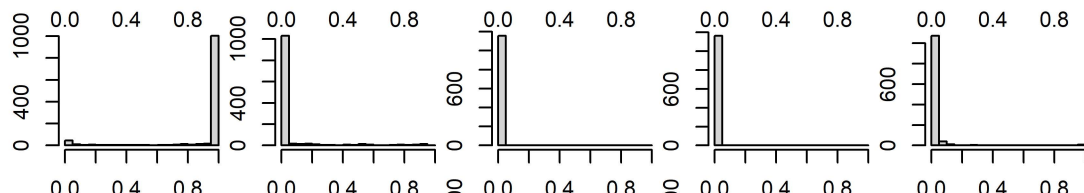

AC

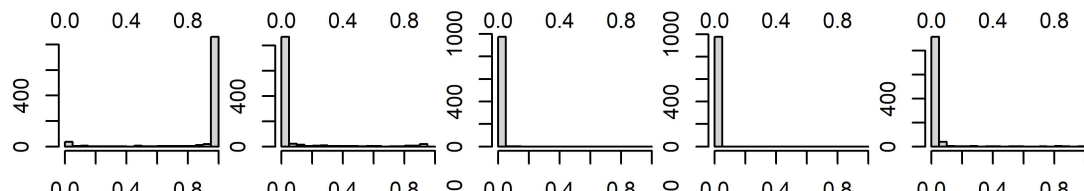

AT

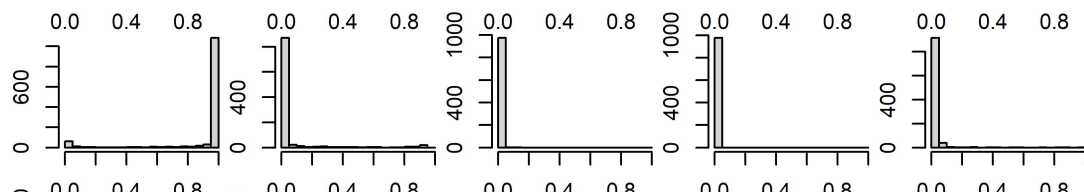

HAA

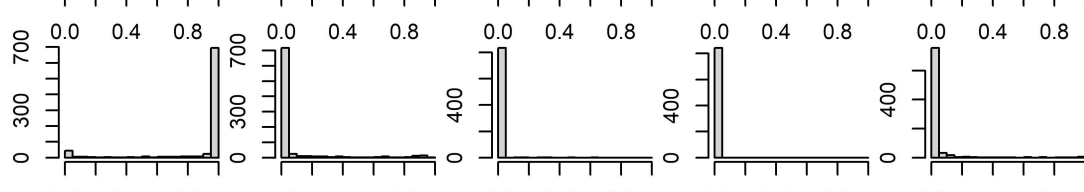

HLV

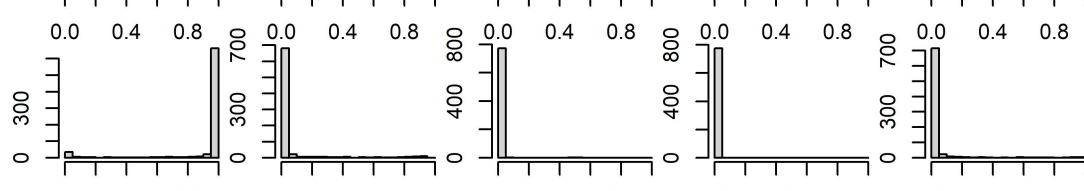

Posterior Probabilities

Supplement: Supplementary file 4 — Additional file 4: Figure S4. Distribution of posterior probabilities (PP0 – PP4) from colocalization analysis with two GWAS phases and five eQTL heart tissues. [file 12863_2021_975_MOESM4_ESM.pdf]

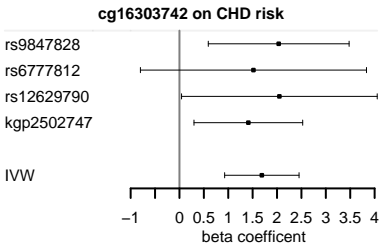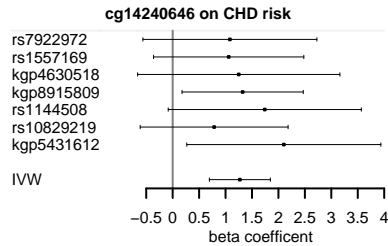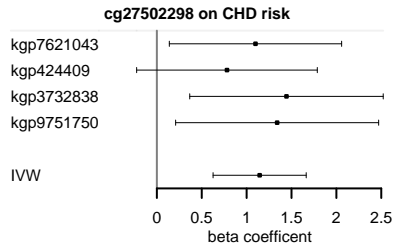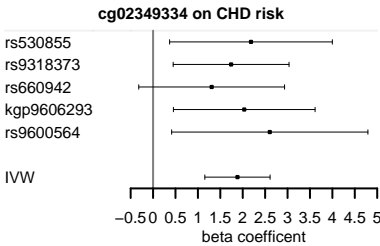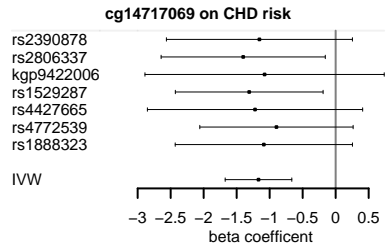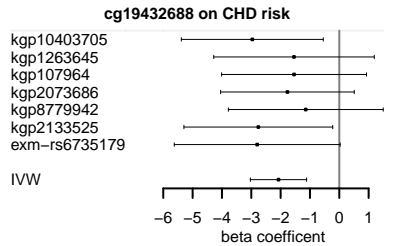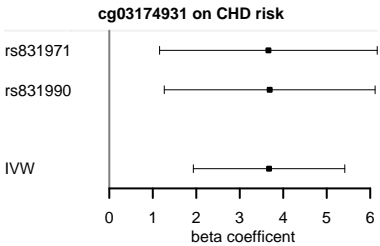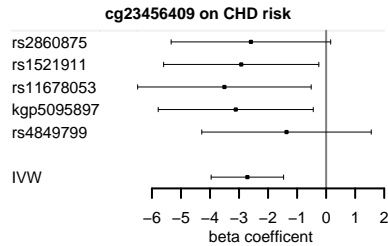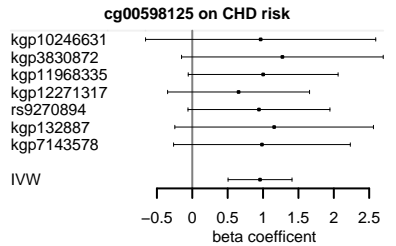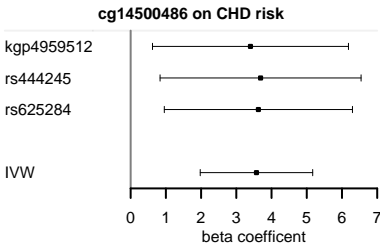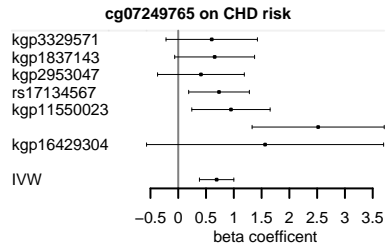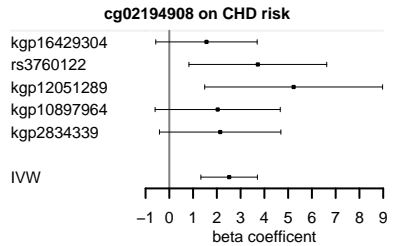

Supplement: Supplementary file 5 — Additional file 5: Figure S5. Two-sample MR for causal effect from mQTL to CHD through CpG. [file 12863_2021_975_MOESM5_ESM.pdf]

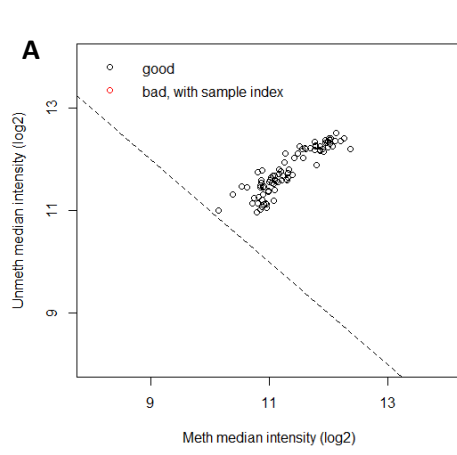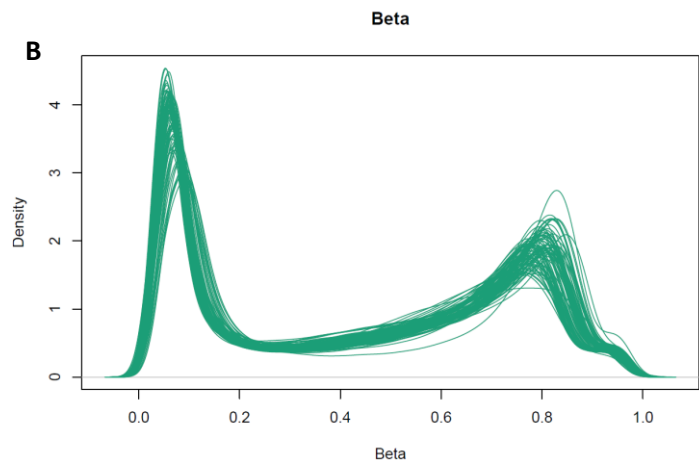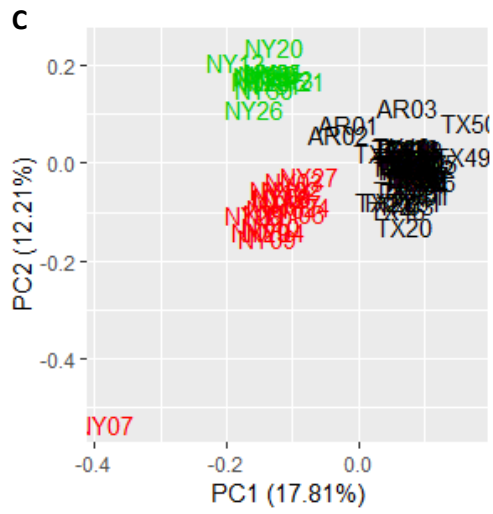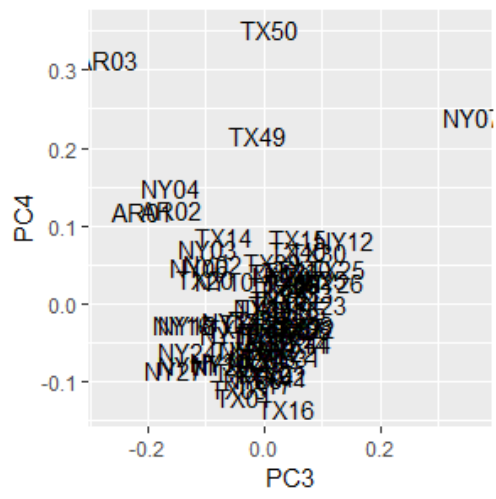

Supplement: Supplementary file 6 — Additional file 6: Figure S6. Sample QC. A. Median intensities for methylated channels vs unmethylated channels. No bad quality sample was identified. B. Distribution of methylation beta values across samples. One sample (internal ID: NY07) showed abnormal distribution. C. Principal components of Epigenomic profiles. Red color: fetal heart samples; Green color: adult heart samples; Black color: ages unknown. One sample (NY07) was removed from the analysis. [file 12863_2021_975_MOESM6_ESM.pdf]
